# Supplementary material for: Longitudinal prevalence and co-carriage of pathogens associated with nursing home acquired pneumonia in three long-term care facilities
Source: PLOS Glob Public Health. 2025 Aug 22;5(8):e0004954. doi: 10.1371/journal.pgph.0004954 (PMC12373211; doi:10.1371/journal.pgph.0004954)
Supplement: Attachment — Submitted filename: Reviewer_response.docx [file pgph.0004954.s006.docx]

Dear Ben Pascoe,

We would like to thank you and the reviewers for the time and effort spent evaluating our manuscript titled: “*Longitudinal prevalence and co-carriage of pathogens associated with nursing home acquired pneumonia in three long-term care facilities”*. We appreciate the constructive feedback, which has significantly improved the quality of this work.

In the revised manuscript we have addressed all comments provided by you and the reviewers and made the suggested changes. Below, we provide a point-by-point response to each comment in red text. The changes in the revised manuscript have been tracked.

Thank you again for considering our revised manuscript.

Sincerely,

Tara N. Furstenau, Ph.D.

Assistant Research Professor

School of Informatics, Computing, and Cyber Systems

Northern Arizona University

Flagstaff, Arizona

Tara.furstenau@nau.edu

## Reviewer 1

Weaknesses:

- Generalizability: Study is limited to LTCFs in one geographic region, potentially affecting broader applicability.
- Sampling Scope: Only two body sites were sampled, which might underestimate overall colonization.
- Pathogen Resolution: Lack of strain-level genotyping limits understanding of intra-species variability.
- Detection Limitations: The absence of *Chlamydia pneumoniae* detection is noted but not fully explored.

Suggestions for Improvement:

- Expand Geographic Scope: Consider including additional LTCFs from diverse regions to enhance generalizability.
- Broaden Sampling: Incorporate additional anatomical sites (e.g., throat, skin) to capture a more complete picture of colonization.
- Strain-Level Analysis: Integrate genotyping or whole-genome sequencing to distinguish strain-specific dynamics.
- Address Negative Findings: Provide further discussion or additional experiments to clarify the non-detection of *Chlamydia pneumoniae*.
- Clarify Limitations: Expand on potential sampling biases and confounding factors and discuss how these might influence the results.

We thank the reviewer for their thoughtful comments about the strengths and weaknesses of our study. We have added a paragraph to the discussion section explicitly addressing the study’s limitations, including geographic scope, sampling sites, strain-level analysis, non-detection of *C. pneumoniae*, and potential confounding factors.

## Reviewer 2

Line [120] [-160]: consider putting the manufacturer’s address of the assays used from them.

We included the manufacture and address of the primers and probes used in the assay.

Line [138-140]: Potentially mention briefly how there were adjustments made for the oligonucleotide sequences or provide references that do.

We have expanded on the methods for the oligonucleotide adjustments both in the text and in the caption for the supplemental table.

Consider making figure 3 a bit bigger to see the left-side figure.

We have split up the original figure and enlarged the left panel to improve legibility.

## Reviewer 3

Lines 253–259: For the results regarding the factors most strongly influencing bacterial load, please include a figure or table to clearly illustrate the data.

A table summarizing the significant predictors of bacterial load has been added to the results section.

Were there any changes in sampling methods or study protocols over the two-year observation period?

There were no changes to the protocol during the study. We have added a statement confirming this in the discussion.

Additionally, please discuss the differences between persistent and transient colonizers, as well as the specific niches each pathogen predominantly colonized. Consider addressing how the biological characteristics of these bacteria may explain their colonization patterns.

We have added a more detailed discussion of colonization duration and niche preferences for each pathogen, supported by relevant literature on their biological characteristics.

## Reviewer 4

The difference between micro- and macro-aspiration as mentioned in the introduction should be better explained.

We have revised the introduction to expand on the definitions.

No information is provided on co-morbidities or medication use, which is a significant omission, as these factors can strongly influence mucosal immunity and, consequently, the likelihood of colonization

We added background on the importance of comorbidities and medication use in colonization risk and acknowledged this as a limitation due to unavailable participant-level data in the discussion

Related to the question above the presence (‘colonization’) of *Pseudomonas aeruginosa* in the upper respiratory tract is generally rare. *P. aeruginosa* is an environmental species and is omnipresent, it is generally known to be a contaminant. Looking at the ‘colonization’ pattern for this species in figure 3, it looks very ‘patchy’, this suggests that contamination may be involved.

We have added the following paragraph to the discussion to address this concern “The relatively high detection rate of *P. aeruginosa* warrants careful interpretation, as this organism is not a typical colonizer of the upper respiratory tract in healthy humans and is ubiquitous in the environment. Although several participants exhibited repeated positive detections over multiple weeks — suggesting true establishment of colonization in some cases — many detections occurred at only a single time point. Although we included non-template controls throughout extraction and amplification steps to rule out technical contamination, the sporadic carriage pattern raises the possibility that some detections may reflect environmental contamination or short-lived exposure rather than truly established colonization. Further investigation is needed to determine whether *P. aeruginosa* can consistently establish colonization in this population or whether its detection in upper respiratory samples reflects exposure to environmental sources.”

It is unclear why the authors chose to collect oral swabs, in addition to nasal swabs, rather than the more commonly used oropharyngeal and nasopharyngeal swags seen in most studies. This methodological choice has likely led to an underestimation of certain bacterial species, particularly *Streptococcus pneumoniae.*

We have added justification for our choice of nasal and oral swabs in the methods section. These sites were selected to ensure tolerability and compliance during the routine longitudinal sampling of elderly participants. We expanded the discussion of other common sampling sites and point out that carriage rates that we report are similar if not higher to previous findings so we do not expect that sampling these sites significantly underestimated carriage.

On page 9, line 236: I would propose to replace the word ‘continuous’ by ‘persistent’.

Here we are referring to the duration of consecutive positive detections, not just in persistent carriers. We have clarified the statement in the text as “The average duration of a colonization episode — defined as a continuous stretch of consecutive positive detections at either of the two body sites — was approximately … “

On page 9, line 241-242, the authors state ‘…. while *H. influenzae* and *P. aeruginosa* typically appear transiently’. I disagree with this statement, as mentioned *P. aeruginosa* is generally found in immunocompromised or individuals with chronic lung disease and in those individuals, it is able to colonize the lungs (not particularly) furthermore I am not sure whether transient colonization is typical for *H. influenzae*, I would like to the see evidence from the literature.

We have expanded the literature review around these points to provide context for *P. aeruginosa* and *H. influenzae* colonization.

Page 10, paragraph 253-259, it is unclear to which figure/result this text refers.

We have included a table of results that were referenced in this section.

Page 10, lines 262-266, ‘However, the co-colonization rates did not appear to be more frequent than what is expected by change (p>0.4, under the assumption of independence of colonization events).’ This is not clearly explained, based on what do the authors assume independent colonization events? The highest frequency of co-colonization is with *S. pneumoniae* and *H. influenzae* (confirming what is quite well known from the literature), it seems to me that this is more frequent that any other combination.

We have expanded on the methods and results sections to clarify this result. While there was a high frequency of co-colonizing events, this analysis was used to assess whether observed co-colonization rates differed from expectations under independence (given the marginal positivity rates) to see if this was random or if there was evidence of positive or negative associations between the pathogens.

Page 11, line 271, ‘multiple bodies sites’ is confusing, and should be more specifically called ’nasal and oral cavity’

This has been updated

In the Discussion, there is a noticeable lack of references to studies that have examined colonization of these species in similar age groups. The authors could have included a brief section on age-related differences in colonization patterns, as well as addressed the unexpectedly high prevalence of *Pseudomonas aeruginosa*. Overall, the Discussion does not sufficiently engage with existing literature and fails to contextualize the findings within what was already known prior to this study.

We have substantially expanded the literature review in the discussion section to better contextualize the findings of the study.
